# Supplementary material for: Male sex identified by global COVID-19 meta-analysis as a risk factor for death and ITU admission
Source: Nat Commun. 2020 Dec 9;11:6317. doi: 10.1038/s41467-020-19741-6 (PMC7726563; doi:10.1038/s41467-020-19741-6)
Supplement: Supplementary file 1 — Supplementary Information [file 41467_2020_19741_MOESM1_ESM.pdf]

GLOBAL COVID-19 META-ANALYSIS IDENTIFIES MALE SEX AS A RISK FACTOR FOR ITU AND DEATH.

Hannah Peckham, Nina M. de Gruijter, Charles Raine, Anna Radziszewska, Coziana Ciurtin, Lucy R. Wedderburn, Elizabeth C. Rosser, Kate Webb* and Claire T. Deakin*

*Corresponding Authors: kate.webb@uct.ac.za, c.deakin@ucl.ac.uk

Supplementary Material

1. **Supplementary Data 1**
   (separate Excel document: 20-09-23 Sex Bias in COVID-19 Supplementary Data.xlsx)
2. **Supplementary Analysis**
   a. **Methods and results of sensitivity analysis to evaluate bias**
   b. **Discussion of sensitivity analysis to evaluate bias**
   c. **Supplementary Table 1: Comparison of Random Effect Estimates With and Without "Trim-and-fill"**
   d. **Supplementary Figure 1. Funnel plots representing effect estimates and standard errors of each report contributing to meta-analysis.**
3. **References for Supplementary Material**

Reproducible R code for replicating all results is available at https://github.com/claire-deakin/covid19-sex-bias/blob/main/SexBiasCovid19.R and at http://doi.org/10.25375/uct.12952151

The code requires the CSV file COVID Sex Bias Table 25-09-2020.csv, which is available at https://github.com/claire-deakin/covid19-sex-bias/blob/main/COVID%20Sex%20Bias%20Table%2025-09-2020.csv and at http://doi.org/10.25375/uct.12952151.

This CSV file contains identical data to the Supplementary Data 1 (20-09-23 Sex Bias in COVID-19 Supplementary Data.xlsx).

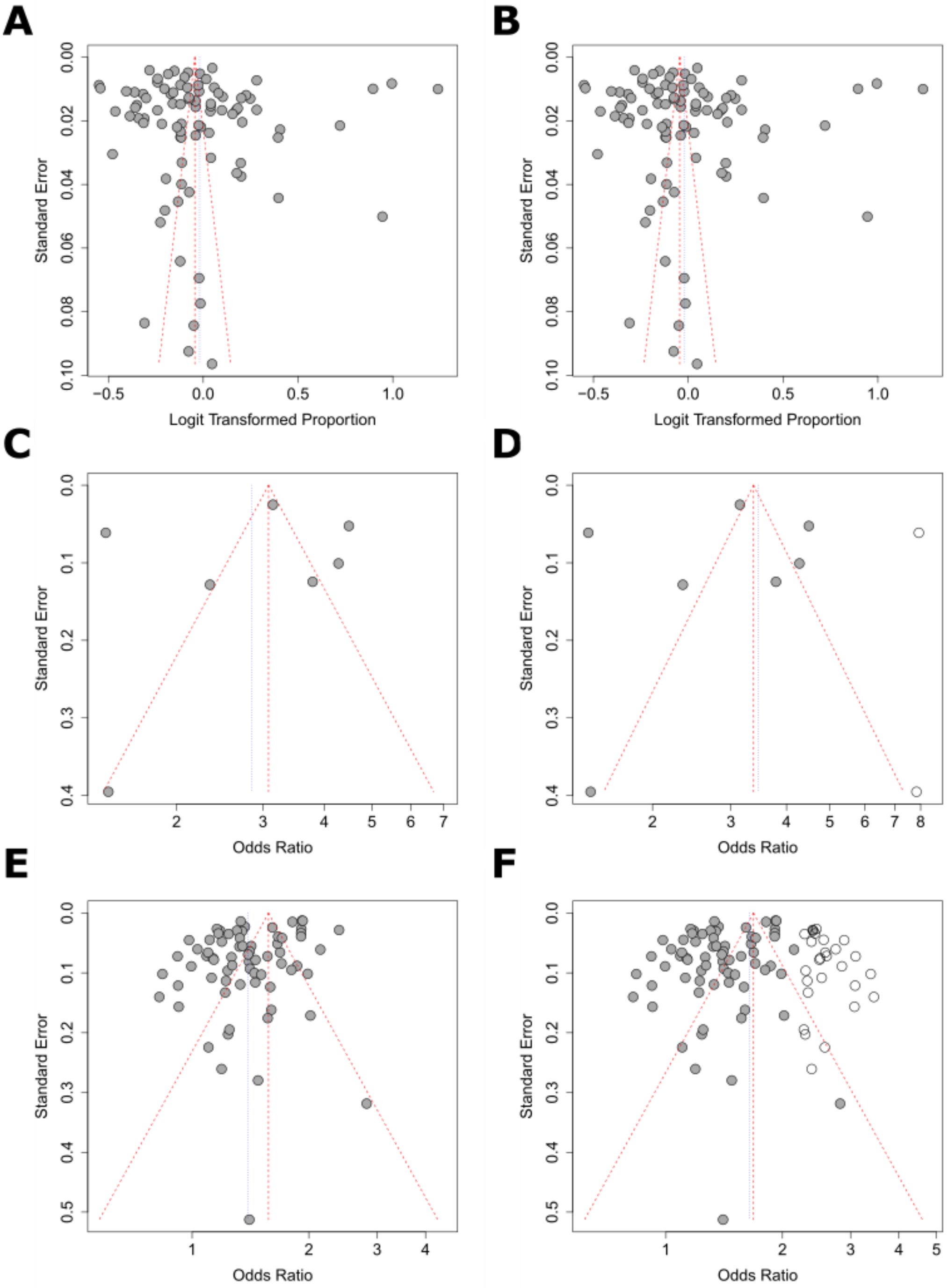

Supplementary Figure 1. Funnel plots representing effect estimates and standard errors of each report contributing to meta-analysis. Funnel plots for estimated **(A)** proportion of male infections, **(B)** proportion of male infections with additional imputed values following “trim-and-fill”, **(C)** odds ratios for association of intensive unit admission with male sex, **(D)** odds ratios for association of intensive unit admission with male sex with additional imputed values following “trim-and-fill”, **(E)** odds ratios for association of mortality with male sex, **(F)** odds ratios for association of mortality with male sex following “trim-and-fill”. Grey circles represent values for reports included in each analysis. White circles in (B) and (D) represent imputed values added by “trim-and-fill” for sensitivity analysis of possible bias. Red lines represent fixed effect estimates and blue lines represent random effect estimates. Sides of the triangle illustrate the expected inverted funnel shape.

Supplementary Table 1: Comparison of Random Effect Estimates With and Without "Trim-and-fill"

Analysis	Estimate	95% Confidence Interval	P-value
Proportion of male infections	0.50	0.48-0.51	0.56
Proportion of male infections with "trim-and-fill"	0.50	0.48-0.51	0.56
Odds ratio for association of male sex with ITU admission	2.84	2.06-3.92	1.9e-10
Odds ratio for association of male sex with ITU admission with "trim-and-fill"	3.46	2.43-4.93	5.9e-12
Odds ratio for association of male sex with mortality	1.39	1.31-1.47	5.0e-30
Odds ratio for association of male sex with mortality with "trim-and-fill"	1.64	1.55-1.74	8.8e-66

2.a. Methods and results of sensitivity analysis to evaluate bias

Reporting bias, possibly due to selective reporting of outcomes and analysis and biases towards certain locations, is a recognised challenge for meta-analyses.[1] In this study, biases may result from the absence of summary data on confirmed infections, ITU admission and mortality broken down by sex.

To evaluate the possibility of reporting bias influencing our results, funnel plots and sensitivity analyses were performed. Visual inspection of funnel plots did not indicate obvious asymmetry for the analyses of proportion of male infections (Supplementary Figure 1A), and odds ratios for ITU admission (Supplementary Figure 1C) and mortality (Supplementary Figure 1E). Funnel plots were produced using the "meta" package (R code below in Supplementary material).

Formal tests of bias using the weighted linear regression method (Egger's test) did not indicate the presence of bias in our analyses of proportion of male infections or ITU admission (p=0.51 and p=0.78, respectively), but suggested bias may be a concern in our analysis of mortality (p=0.0013).[2] However, these tests have low power and can lead to incorrect conclusions about lack of bias when study numbers are low,[1] such as our analysis of ITU admissions. Therefore, and given the result of the test for bias for our mortality analysis, sensitivity analyses were performed using the "trim-and-fill" method.[3] "Trim-and-fill" was performed using the "meta" package. The output of this function includes the updated random effect estimates.

Funnel plots following "trim-and-fill" were produced for the analyses of proportion of male infections (Supplementary Figure 1B), and odds ratios for ITU admission (Supplementary Figure 1D) and mortality (Supplementary Figure 1F).

Similar estimates were obtained when possible biases were accounted for in this manner (Supplementary Table 2). Although the estimated proportion of male infections following "trim-and-fill" differed statistically from the hypothesised null of 0.5, it was almost identical to the proportion estimated without "trim-and-fill". Although the estimated odds ratios for ITU admission and mortality were not identical following "trim-and-fill", the direction of the estimates was the same and both odds ratios were significantly different from the hypothesised null of 1. The estimated odds ratio for ITU admission following "trim-and-fill" was compatible with the 95% confidence interval for the estimate without "trim-and-fill". Interestingly, the estimated odds ratio for mortality is higher following "trim-and-fill" indicating that if selection bias is present, this has resulted in an underestimate of risk.

2.b. Discussion of sensitivity analysis to evaluate bias

While the results of the Egger's test and sensitivity analysis suggest the possibility that reporting bias may have led to an underestimate of the mortality risk for men, we recommend a cautious interpretation of this possibility. Government statistics are an unconventional data source for the application of meta-analysis techniques, which typically use published literature and there are concerns the publication bias towards positive findings leads to overestimates of effect sizes in meta-analyses. Reporting bias or under-estimation of the rates of deaths due to COVID-19 may also be concerns in rapidly-generated government statistics. Future analyses may confirm whether the true odds ratio for mortality in males is closer to 1.39 or 1.64.

References for Supplementary Information

1. Sterne, J. A. C. *et al.* Recommendations for examining and interpreting funnel plot asymmetry in meta-analyses of randomised controlled trials. *BMJ* **343**, (2011).
2. Egger, M., Smith, G. D., Schneider, M. & Minder, C. Bias in meta-analysis detected by a simple , graphical test measures of funnel plot asymmetry. *Bmj* **315**, 629–34 (1997).
3. Duval, S. & Tweedie, R. Trim and fill: A simple funnel-plot-based method of testing and adjusting for publication bias in meta-analysis. *Biometrics* **56**, 455–463 (2000).
