# Supplementary material for: Male sex identified by global COVID-19 meta-analysis as a risk factor for death and ITU admission
Source: Nat Commun. 2020 Dec 9;11:6317. doi: 10.1038/s41467-020-19741-6 (PMC7726563; doi:10.1038/s41467-020-19741-6)
Supplement: Supplementary file 3 — Reporting Summary [file 41467_2020_19741_MOESM3_ESM.pdf]

Corresponding author(s): Dr Kate Webb, Dr Claire Deakin

Last updated by author(s): Sep 27, 2020

Reporting Summary

Nature Research wishes to improve the reproducibility of the work that we publish. This form provides structure for consistency and transparency in reporting. For further information on Nature Research policies, see our Editorial Policies and the Editorial Policy Checklist.

Statistics

For all statistical analyses, confirm that the following items are present in the figure legend, table legend, main text, or Methods section.

n/a	Confirmed	
☐	☒	The exact sample size (*n*) for each experimental group/condition, given as a discrete number and unit of measurement
☐	☒	A statement on whether measurements were taken from distinct samples or whether the same sample was measured repeatedly
☐	☒	The statistical test(s) used AND whether they are one- or two-sided<br>*Only common tests should be described solely by name; describe more complex techniques in the Methods section.*
☐	☒	A description of all covariates tested
☐	☒	A description of any assumptions or corrections, such as tests of normality and adjustment for multiple comparisons
☐	☒	A full description of the statistical parameters including central tendency (e.g. means) or other basic estimates (e.g. regression coefficient) AND variation (e.g. standard deviation) or associated estimates of uncertainty (e.g. confidence intervals)
☐	☒	For null hypothesis testing, the test statistic (e.g. *F*, *t*, *r*) with confidence intervals, effect sizes, degrees of freedom and *P* value noted<br>*Give P values as exact values whenever suitable.*
☒	☐	For Bayesian analysis, information on the choice of priors and Markov chain Monte Carlo settings
☐	☒	For hierarchical and complex designs, identification of the appropriate level for tests and full reporting of outcomes
☒	☐	Estimates of effect sizes (e.g. Cohen's *d*, Pearson's *r*), indicating how they were calculated

Our web collection on statistics for biologists contains articles on many of the points above.

Software and code

Policy information about availability of computer code

Data collection	No software was used for data collection.
Data analysis	Data were analysed using R version 3.6.1 and the "meta" package 4.11-0. Code used for the analysis is available with supplementary materials and has been deposited at http://doi.org/10.25375/uct.12952151.

For manuscripts utilizing custom algorithms or software that are central to the research but not yet described in published literature, software must be made available to editors and reviewers. We strongly encourage code deposition in a community repository (e.g. GitHub). See the Nature Research guidelines for submitting code & software for further information.

Data

Policy information about availability of data

All manuscripts must include a data availability statement. This statement should provide the following information, where applicable:

- Accession codes, unique identifiers, or web links for publicly available datasets
- A list of figures that have associated raw data
- A description of any restrictions on data availability

All data contributing to this analysis are provided in Supplementary Table 1 and published at figshare (http://doi.org/10.25375/uct.12952151). Screenshots of all sources from date of access are archived and may be available on request.

Field-specific reporting

Please select the one below that is the best fit for your research. If you are not sure, read the appropriate sections before making your selection.

☒ Life sciences ☐ Behavioural & social sciences ☐ Ecological, evolutionary & environmental sciences

For a reference copy of the document with all sections, see nature.com/documents/nr-reporting-summary-flat.pdf

Life sciences study design

All studies must disclose on these points even when the disclosure is negative.

Sample size	This study is a meta-analysis of 90 reports describing the sex of 3,111,714 COVID cases in 46 countries. No power calculation performed; we sought to include data representing as many individuals as possible in this analysis.
Data exclusions	Reports were included if they contained sex as a variable in data describing case number, intensive treatment unit (ITU) admission, or mortality. Reports were excluded if the case numbers were low (<5), if they were thought to represent the same individuals or if they did not contain sufficient data on the variables required for analysis e.g. ITU admission by sex or mortality by sex.
Replication	This is a non-experimental study, so not applicable. However, as the numbers of the cases increased substantially during the course of peer review and data from more jurisdictions became available, we updated the numbers from the analysis that was originally submitted. The estimates obtained with updated number were very similar to the estimates in the original analysis.
Randomization	Not applicable; this is an observational study of reported summary data with no treatment allocation.
Blinding	Blinding not relevant to this study; this is a report of confirmed COVID-19 cases intensive care admission and mortality broken down by sex.

Reporting for specific materials, systems and methods

We require information from authors about some types of materials, experimental systems and methods used in many studies. Here, indicate whether each material, system or method listed is relevant to your study. If you are not sure if a list item applies to your research, read the appropriate section before selecting a response.

Materials & experimental systems

n/a	Involved in the study	
☒	☐	Antibodies
☒	☐	Eukaryotic cell lines
☒	☐	Palaeontology and archaeology
☒	☐	Animals and other organisms
☐	☒	Human research participants
☒	☐	Clinical data
☒	☐	Dual use research of concern

Methods

n/a	Involved in the study	
☒	☐	ChIP-seq
☒	☐	Flow cytometry
☒	☐	MRI-based neuroimaging

Human research participants

Policy information about studies involving human research participants

Population characteristics	Data represent the full age range of COVID-19 cases. Details of individuals' genotypes, treatment or diagnoses with conditions other than COVID-19 were not available in the source data. Consequently, such covariates could not be controlled for in this analysis
Recruitment	No human research participants were recruited. Data are summary data reported by individual countries. We sought to represent as many global regions as possible. We cross-checked against countries listed on "Worldometer" as reporting case numbers by sex, in order to ensure global representativeness.
Ethics oversight	Ethics oversight is not required. This is an analysis summary data made publicly available on the internet by mostly government sources. No individual-level data contributed to this analysis.

Note that full information on the approval of the study protocol must also be provided in the manuscript.
