# Supplementary material for: Male sex identified by global COVID-19 meta-analysis as a risk factor for death and ITU admission
Source: Nat Commun. 2020 Dec 9;11:6317. doi: 10.1038/s41467-020-19741-6 (PMC7726563; doi:10.1038/s41467-020-19741-6)
Supplement: Supplementary file 4 — Description of Additional Supplementary Files [file 41467_2020_19741_MOESM4_ESM.pdf]

Description of Additional Supplementary Files

File Name:
20-09-23 Sex Bias in COVID-19 Supplementary Data.xlsx

Description:
Supplementary Data 1, spreadsheet of all data used for meta-analyses
Data are available at http://doi.org/10.25375/uct.12952151
